# Effect of different breathing frequencies with breath-holding on muscle activity and coordination during butterfly swimming in national-level female swimmers

**DOI:** 10.3389/fspor.2025.1613966

**Published:** 2025-07-24

**Authors:** Keisuke Kobayashi Yamakawa, Yasuo Sengoku, Hideki Takagi

**Affiliations:** Institute of Health and Sport Sciences, University of Tsukuba, Tsukuba, Ibaraki, Japan

**Keywords:** competitive swimming, performance, motion analysis, electromyography, muscle synergy analysis

## Abstract

The aim of this study was to clarify the effect of different breathing frequencies with breath-holding on muscle activity and coordination during butterfly swimming in competitive swimmers. Eight national-level female swimmers participated in this study. They performed 25-m maximal butterfly swims with two breathing frequencies (task 1: swimming with the frontal breathing action for every stroke, and task 2: swimming while alternating the cycles with frontal breathing action and with breath-holding). From these tasks, the three different cycles (breathing cycle in task 1, breathing and breath-holding cycles in task 2) were analyzed. Surface electromyography was measured from 12 muscles of the right upper and lower limb, and trunk. A nonnegative factorization algorithm was used for muscle synergy analysis from the electromyographic data. Our results showed the activity timing for Triceps brachii, Deltoideus anterior, Latissimus dorsi, Biceps femoris and Gastrocnemius became earlier in the breath-holding cycle compared to those in the breathing cycle of task 1. However, the activity timing of all the muscles did not change between the breathing and breath-holding cycles of task 2. The number of muscle synergies was the same across the three cycles, except for one swimmer. The muscle combination of all the synergies was very similar across the three cycles. In contrast, the drive timing of the two synergies, which relate to the arm-pull movement and the first and second upward kicks, respectively, became earlier in the breath-holding cycle compared to those in the breathing cycle of task 1, while the drive timing did not change between the breathing and breath-holding cycles of task 2. These results suggest that the temporal characteristics of muscle activity and synergies are more influenced by different breathing frequencies than by frontal breathing action. Therefore, researchers should consider these effects when analyzing muscle activity during butterfly swimming.

## 1 Introduction

Breathing action is necessary to continue swimming and is one of the basic aquatic skills. In butterfly stroke, competitive swimmers have used two breathing styles (frontal breathing and lateral breathing). Most current swimmers seem to use frontal breathing style, as seen in recent competitions. For example, at the 2024 Paris Olympics, all of the finalists in the men's and women's 100 m and 200 m butterfly events used the frontal breathing style. In frontal breathing style, competitive swimmers lift their head, shoulders, and trunk toward the surface during the late half of underwater arm stroke to inhale (1). This action causes an increase in the angle of body inclination (2); therefore, it has been discussed whether breathing action causes a reduction in performance (1–3). In addition, Seifert et al. (4) reported that the frontal breathing action during butterfly swimming reduced the continuity of arm and leg propulsive actions during sprint paces. Moreover, Barbosa et al. (5) reported that horizontal hand velocity during the out-sweep phase was higher in breath-holding cycles than in cycles with forward breathing, suggesting that propulsive force may be generated earlier in the stroke with breath-holding. These findings support the possibility that breathing reduces swimming speed, while breath-holding increases it. In fact, when observing the breathing frequency during the first 50 m of the men's and women's 100-m butterfly finals at the 2024 Paris Olympics, four swimmers breathed once per stroke, 11 swimmers breathed once every two strokes, and one swimmer breathed twice every three strokes. Accordingly, the typical breathing frequency for many swimmers is once every two strokes (4), and such swimmers perform the swimming movement without breathing action (breath-holding), which has the potential to increase swimming speeds (3), during butterfly stroke races. Nevertheless, it can be observed that some swimmers breathe with each stroke, even at international level.

Surface electromyography (EMG) has been used to assess neuromuscular activity in sports movements and to clarify the mechanisms for achieving the technique in terms of muscle activity and neural control (6). However, a review of swimming EMG studies indicated that none of the studies performed on swimming focused on the influence of the breathing action on skeletal muscles (7). Therefore, it was unclear how the breathing action affects muscle activity. Furthermore, recent swimming EMG studies have conducted muscle synergy analysis (8–10). This analysis is a method for quantifying motor control by using multi-channel EMG recordings (11). Muscle synergy is defined as the synchronous activation of a group of muscles, and muscle synergy analysis extracts multiple muscle synergies from a movement, revealing the underlying mechanisms of the muscular coordination required to control the movement (9). Previous studies on butterfly swimming have revealed that four muscle synergies were extracted from the movement during butterfly swimming in swimmers with different history of shoulder injury or with different performance levels (8, 9). This result indicates that swimmers perform the butterfly stroke movements using a small number of muscle synergies. However, these results were obtained by analyzing the movements that the swimmers breathed frontally with each cycle. Investigating the effects of breathing action and frequency on muscle activity and coordination would provide clearer evidence to help researchers exclude these effects. Therefore, the aim of this study was to clarify the effect of different breathing frequencies with breath-holding on muscle activity and coordination during butterfly swimming in competitive swimmers. We hypothesized that the intensity of muscle activity increases during the breathing cycle as swimmers need to lift their body to inhale, and that the cooperation between the upper and lower limb muscles in the muscle synergy increases in the breath-holding cycle as the continuity of arm and leg propulsive action improves.

## 2 Materials and methods

### 2.1 Participants

Eight female competitive swimmers (age: 19.6 ± 0.7 years, height: 1.61 ± 0.04 m, and body mass: 55.9 ± 4.3 kg, years of experience as a competitive swimmer: 9.6 ± 0.7 years) participated in this study. As differences in swimmer's performance levels affect butterfly stroke movement (12), we decided to include only competitive swimmers classified as Tier 3 (national level) according to the classification of McKay et al. (13). As the exclusion criteria, swimmers who were unable to perform at their best in the butterfly stroke due to an injury or illness were excluded from this study. All participants belonged to a university swimming club and had undergone training on swimming 6,000–10,000 m per day, six days a week. Their specialties included four butterfly, one backstroke, one freestyle, and two individual medley swimmers. All participants preferred to use the frontal breathing style for butterfly swimming. Their average personal best time in the 100 m short-course butterfly was 61.97 (±1.37) s, which was 87.3 (±0.19) % of the world record. All participants were fully informed of the risks, benefits, and stressors of the study and written informed consent was obtained from all participants. The study was conducted in accordance with the Declaration of Helsinki and approved by the University Research Ethics Committee (2021-4).

### 2.2 Swim trials

The experiment was conducted in a 25-m indoor pool that had a water depth of 1.35 m and a water temperature of 28 degrees Celsius. The participants performed a self-determined warm-up for 30 min before the swim trials. After the warm-up, they performed 25-m butterfly swims with two breathing frequencies: (1) swimming with the breathing action for every stroke and (2) swimming while alternating the cycles with and without breathing. The selection of these tasks was based on the description in a previous study by Seifert et al. (4) that typical swimmers breathe once every two arm strokes or once per stroke. For these tasks, participants were instructed to breathe using a frontal style and to accelerate from the push-off start in order to cover 12.5 m, followed by a maximum effort swim from 12.5 to 25 m. The participants performed these trials twice in random order, with a 3-min rest period. The following three cycles were analyzed from a total of four trials: (1) a breathing cycle in task 1 (BE); (2) a breathing cycle in task 2 (BABH); and (3) a breath-holding cycle in task 2 (BH).

### 2.3 Kinematic analysis

**Figure 1:**
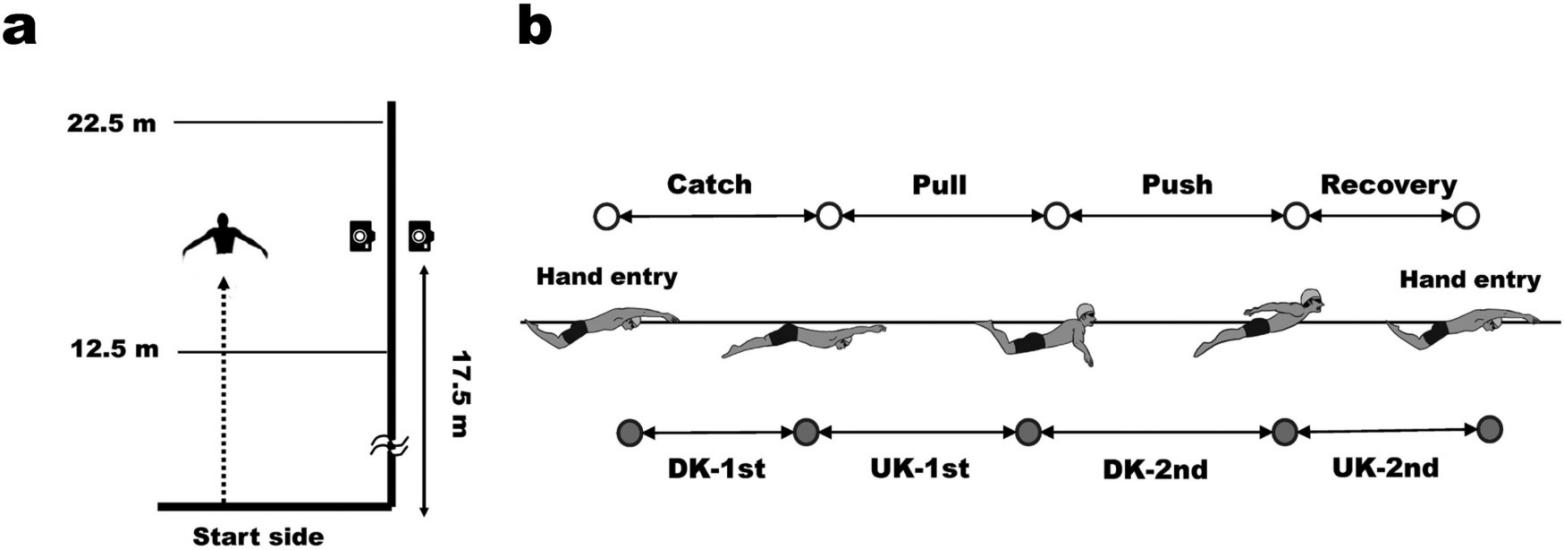
Experimental setting **(a)** and definition of arm stroke and leg kick phases in butterfly swimming **(b)**.

The sagittal movements during the trials were recorded at a sampling rate of 60 Hz using a synchronous above-water and underwater video-recording system (Figure 1a). This system was placed at 17.5 m from the start wall and recorded the video ranging from 12.5 to 22.5 m. As a body landmark, an LED marker was attached to the greater trochanter of the femur (hip) of each swimmer. The hip coordinates were digitized using a motion analysis software (Tracker, Open-Source-Physics), and the digitised coordinates were converted into global coordinates using the 2D-DLT method.

In this study, one stroke cycle was defined as the period from the right-hand entry to the next-hand entry. The arm stroke and leg kick movements were divided into four phases, as shown in Figure 1b, based on the definitions provided by Chollet et al. (14). The stroke rate (SR, cycles/min), stroke length (SL, m/cycle), and relative duration (%) of the arm and leg phases were calculated from the video and hip coordinates. Swimming velocity (V, m/s) was defined as the horizontal hip velocity, and the mean, maximum, and minimum swimming velocities (V_mean,_ V_max_, and V_min_, respectively) were calculated from the hip coordinates. The intra-cycle variation in swimming velocity (dV) was calculated using Equation 1:

(1) $$dV\;(\text{\%})=\;\frac{\sqrt{\sum_{i}{\left(V_{i}-V_{mean}\right)}^{2}F_{i}/n}}{\sum_{i}V_{i}F_{i}/n}\times 100$$

where V_i_ represents the instant swimming velocity, Fi represents the absolute frequency, and *n* represents the number of observations. This parameter is related to the energy cost of butterfly swimming (15). The averages of these parameters from the data of 2–4 cycles were used for statistical analysis.

### 2.4 EMG analysis

Surface EMG was measured using a wireless system with a data logger (BioLog System, S&ME Inc., Japan) at a sampling rate of 1,000 Hz. EMG signals were collected using dry electrodes with an interelectrode distance of 0.02 m. The measured muscles were the following twelve muscles: Biceps brachii (BB), triceps brachii (TB), anterior deltoid (DA), posterior deltoid (DP), pectoralis major (PM), latissimus dorsi (LD), rectus abdominis (RA), erector spinae (ES), rectus femoris (RF), biceps femoris (BF), tibialis anterior (TA), and medial head of gastrocnemius (GAS). The electrode locations were determined according to Yamakawa et al. (9), as shown in Table 1. Before the electrodes were fixed, the skin surface was shaved, abraded, and cleaned with alcohol. According to the methodology of Kobayashi et al. (16), The attached EMG sensors were waterproofed by covering them with water-resistant tape.

**Table 1:** Muscle abbreviations and electrode placement.

Muscle name	Electrode placement
Biceps brachii (BB)	On the line between the medial acromion and the fossa cubit at 1/3 from the fossa cubit
Triceps brachii (TB)	50% on the line between the posterior crista of the acromion and the olecranon at 2 finger widths medial to the line
Deltoideus anterior (DA)	One finger width distal and anterior to the acromion
Deltoideus posterior (DP)	Center the electrodes in the area about two finger breaths behind the angle of the acromion
Pectoralis major (PM)	2 cm below the clavicle, precisely medial to the axillary fold
Latissimus dorsi (LD)	The oblique angle over the LD muscle, approximately 4 cm below the inferior tip of the scapula and midway between the spine and lateral edge of the torso
Rectus abdominis (RA)	3 cm lateral to the umbilicus
Erector spinae (ES)	Two finger width laterals from the proc. spin. of L1
Rectus femoris (RF)	50% on the line from the anterior spina iliac superior to the superior part of the patella
Biceps femoris (BF)	50% on the line between the ischial tuberosity and the lateral epicondyle of the tibia
Tibialis anterior (TA)	1/3 on the line between the tip of the fibula and the tip of the medial malleolus
Gastrocnemius medialis (GAS)	On the most prominent bulge of the muscle

The placements of electrodes were determined according to the SENIAM project (http://www.seniam.org) and as described in a previous study (9).

Using the MATLAB software (version R2023b, MathWorks Inc.), the raw EMG data were filtered using a bandpass filter between 20 and 500 Hz. The filtered EMG data were rectified and then passed through a low-pass filter (12-Hz, fourth-order Butterworth) to derive the EMG envelopes. The EMG envelopes were interpolated to 101 percentiles for time normalization, and the mean ensemble curves were created using the data of 2–4 cycles. The EMG amplitude was normalized by the peak values during the 5-s exercises of the maximum voluntary isometric contraction (MVC) which was conducted after the swim trials. The averages of the normalized EMG (aEMG) during the stroke cycle were calculated for statistical analysis.

### 2.5 Muscle synergy analysis

The average EMG envelopes were used for muscle synergy analysis. The amplitudes were then renormalized to peak values in a cycle (i.e., 0–1), according to previous studies (8–10). Non-negative matrix factorization (NMF) was performed for muscle synergy analysis, and the NMF was implemented using the algorithm described by Lee and Seung (17). In this procedure, the initial matrix of the EMG data (E) was factorized into the following two components: weighting coefficients or loadings (W), which reflect the combination of muscles, and basic temporal components (C), which correspond to the timing of activation. We performed NMF analysis using Equations 2, 3:

(2) E=WC+e

(3) $$\underset{\begin{array}{c} W>0\\ C>0 \end{array}}{\min}\|E-WC{\|}_{FRO}$$

where E represents the residual error. In Equation 2, E is an m × p initial matrix (m is the number of muscles, and p is the number of time points), W is an m × s matrix, (s is the number of synergies), C is an s × p matrix, and e is an m × p matrix. Equation 3 indicates that matrix E is minimized in Equation 2. The analysis was repeated for each participant, varying the number of synergies between 1 and 11 and selecting the lowest number of synergies that satisfied the criteria of variance accounted for (VAF). The VAF (%) was defined as 100 × the coefficient of determination of the uncentered Pearson's correlation coefficient and was calculated using Equation 4:

(4) $$VAF\;(\text{\%})=\left(1-\frac{\sum_{i=1}^{p}\sum_{j=1}^{n}{\left(e_{i,j}\right)}^{2}}{\sum_{i=1}^{p}\sum_{j=1}^{n}{\left(E_{i,j}\right)}^{2}}\right)\times 100$$

where p = 12, n = 101, and i and j are 1 < i < 12 and 1 < j < 101, respectively. Referring to a previous study (8, 9), s was calculated as the lowest number of synergies with a VAF of >90%.

The extracted synergies in BABH and BH cycles were sorted based on the scalar product (SP) values and those in BE cycle for each participant. The formula for SP is presented in the following sections. After the sorting within a participant, the extracted synergies in BE cycle were sorted based on the values of SP with that in BE cycle of the arbitrary reference participant.

To identify the main muscles contributing to the synergy, a muscle was considered to contribute highly to the synergy if the mean W was above 0.30, referring to a previous study (9). The SP was used to assess the similarity in W between the different cycles. The SP is equal to the uncentered Pearson's correlation and was calculated using Equation 5:

(5) $$SP=\frac{W_{i}^{A}\cdot W_{k}^{B}}{\left\|W_{i}^{A}\|\|W_{k}^{B}\right\|}\;(-1<SP<1)$$

where i and k are the i- (k-) th synergies, respectively. According to Matsunaga et al. (18), it was defined that two W were similar if the SP was >0.75. The similarities of waveform in the individual EMG and C between the different cycles were assessed using the maximum correlation coefficient (*r*_max_) and lag time (%) calculated from the cross-correlation analysis. *r*_max_ corresponds to the maximum similarity of the waveforms, and the lag time corresponds to the time difference in the muscle or synergy activity. According to Oliveira et al. (19), a *r*_max_ above 0.8 was defined as indicating similarity.

### 2.6 Statistical analysis

A priori power analysis using G*Power software (version 3.1.9.7) indicated that a total sample size of five participants would be required to detect a large effect size [Cohen's *f* = 0.73, referring to the minimum effect size of breathing effect reported by Seifert et al. (4)] in repeated one-way analysis of variance (ANOVA) with three measurements, with an alpha level of 0.05 and power of 0.80. Therefore, the sample size of this study was considered sufficient for the statistical analysis.

Statistical analysis was performed using Jamovi software (version 2.6.13, Open source). The normality of all datasets was checked using the Shapiro–Wilk test. If normality was confirmed in the dataset, the parameter was compared between the different three cycles using repeated one-way ANOVA. Then, a *post-hoc* test was performed using Bonferroni's correction. If normality was not confirmed in the dataset, the parameter was compared using Friedman's test. Then, a *post-hoc* test was performed using Wilcoxon signed-rank test. To evaluate the differences in lag time between the different three cycles, a non-parametric one-sample test (Wilcoxon signed-rank test) with zero as the reference value was performed. All statistical significance levels were set at 5%. In addition, eta-squared (*η*^2^) values were used to present the effect sizes for the repeated one-way ANOVA. The threshold values of *η*^2^ that represented small, medium, and large were 0.01, 0.06, and 0.14 (20). The effect sizes (*r*) for the Wilcoxon signed-rank test were calculated by dividing the z-score by the square root of the number of participants. The threshold values of the |*r*| that represented low, medium, and large were 0.1, 0.3, and 0.5 (20).

## 3 Results

Table 2 lists the results of kinematic parameters in the BE, BABH, and BH cycles. The results of the repeated ANOVA confirmed that there was a main effect in the relative durations of the push, recovery and DK-1st phases (all *p* < 0.05, *η*^2^ = 0.09–0.24). The results of the *post-hoc* tests showed that the push phase in the BH cycle was longer than those in the BE and BABH cycles (both *p* < 0.05) and that the recovery phase in the BH cycle was longer than that in the BE cycle (*p* < 0.05).

**Table 2:** Results of the kinematic parameters in the BE, BABH and BH cycles.

Variable, unit	BE	BABH	BH	ANOVA	
				*F*-value	*η* ^2^
V_mean_, m/s	1.50 ± 0.07	1.52 ± 0.05	1.53 ± 0.07	1.13	0.06
V_max_, m/s	1.96 ± 0.20	1.96 ± 0.07	2.08 ± 0.14	1.48	0.14
V_min_, m/s	1.00 ± 0.09	0.99 ± 0.12	1.03 ± 0.10	0.88	0.03
SR, cycle/min	54.4 ± 2.1	55.2 ± 2.1	55.4 ± 2.2	1.63	0.04
SL, m/cycle	1.65 ± 0.08	1.65 ± 0.08	1.65 ± 0.07	0.18	< 0.01
dV (%)	18.1 ± 2.2	17.9 ± 1.6	18.2 ± 1.5	0.10	< 0.01
Catch phase, %	31.1 ± 5.6	30.4 ± 2.4	28.6 ± 4.3	2.57	0.06
Pull phase, %	23.4 ± 3.8	24.1 ± 1.9	22.3 ± 3.1	0.92	0.06
Push phase, %	23.8 ± 2.0	23.2 ± 2.9	25.9 ± 1.7^a^^b^	17.20*	0.24
Recovery phase, %	21.7 ± 2.2	22.3 ± 1.9	23.1 ± 1.6^a^	10.50*	0.09
DK-1st phase, %	18.0 ± 2.3	16.0 ± 1.7	17.3 ± 1.7	4.43*	0.17
UK-1st phase, %	28.7 ± 3.4	30.2 ± 3.0	28.7 ± 1.9	2.78	0.06
DK-2nd phase, %	23.5 ± 2.0	23.1 ± 2.1	22.2 ± 2.2	0.65	0.04
UK-2nd phase, %	29.7 ± 2.8	30.7 ± 2.2	31.5 ± 4.1	2.00	0.06

All data presented as Mean ± SD. An asterisk indicates a significant main effect in a repeated ANOVA, *p* < 0.05.

^a^ Means a significant difference from BE, *p* < 0.05.

^b^ Means a significant difference from BABH, *p* < 0.05.

**Figure 2:**
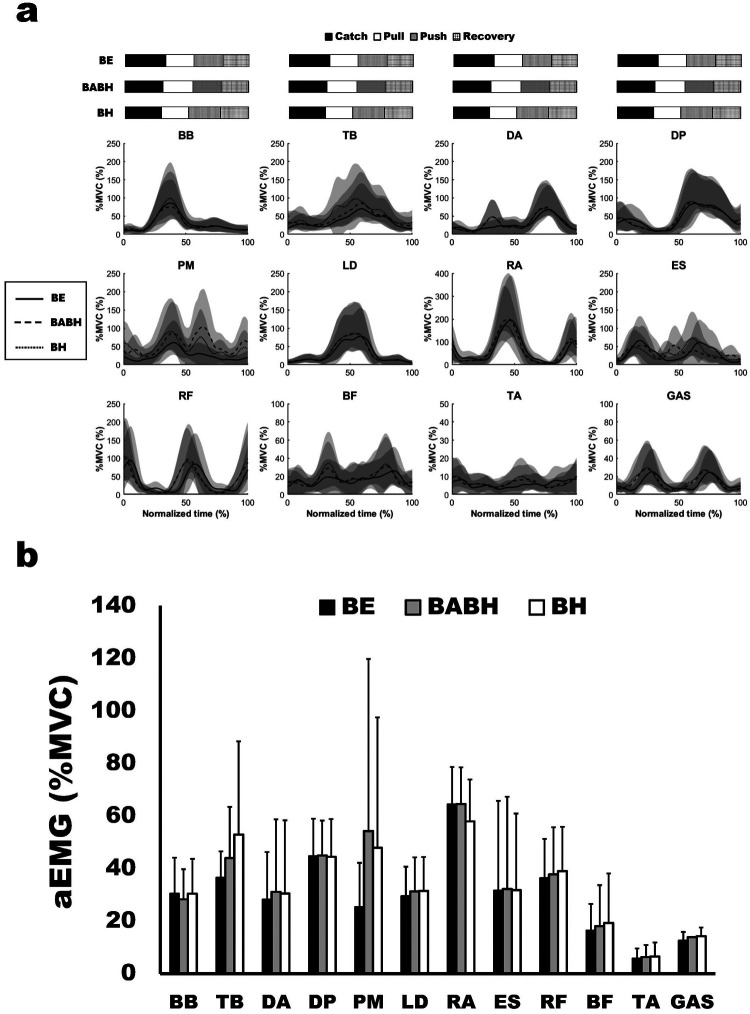
Mean ensemble curves of the normalized EMG for the individual muscles **(a)** and results of aEMG **(b)**. In **(a)**, the solid lines show the results in the BE cycle, the dash lines show the results in the BABH cycle, and the dotted lines show the results in the BH cycle. The horizontal bar graphs show the timing of the average arm stroke phases in each cycle. In **(b)**, the black bars show the results in the BE cycle, the gray bars show the results in the BABH cycle, and the white bars show the results in the BH cycle.

Figure 2a shows the mean ensemble EMG curves for the individual muscles. Table 3 lists the results of the *r*_max_ and lag time of the individual muscle activity between the two different cycles. The mean *r*_max_ was above 0.80 for all the muscles. The results of the one-sample test for the lag time showed that the timing of activity for the DA, LD, RF, BF and GAS in the BABH cycle became earlier than those in the BE cycle (all *p* < 0.05, *r* = 0.72–0.90) and that that the timing of activity for the TB, DA, LD, BF and GAS in the BH cycle became earlier than those in the BE cycle (all *p* < 0.05, *r* = 0.72–0.84). Figure 2b shows the results of aEMG for the individual muscles in the BE, BABH and BH cycles. From the results of the repeated ANOVA and Friedman's test, it was confirmed that there was no main effect on any of the muscles.

**Table 3:** Results of the waveform similarity (r_max_) and lag time for the individual EMG curves.

Variable, unit	BE vs. BABH	BE vs. BH	BABH vs. BH
*r_max_*			
BB	0.98 ± 0.03	0.98 ± 0.02	0.99 ± 0.01
TB	0.97 ± 0.02	0.97 ± 0.03	0.97 ± 0.03
DA	0.93 ± 0.08	0.97 ± 0.03	0.99 ± 0.01
DP	0.97 ± 0.03	0.97 ± 0.03	0.98 ± 0.01
PM	0.92 ± 0.08	0.93 ± 0.11	0.97 ± 0.04
LD	0.99 ± 0.01	0.99 ± 0.01	0.99 ± 0.01
RA	0.95 ± 0.06	0.97 ± 0.02	0.97 ± 0.02
ES	0.97 ± 0.07	0.96 ± 0.06	0.96 ± 0.04
RF	0.91 ± 0.09	0.94 ± 0.04	0.96 ± 0.02
BF	0.96 ± 0.04	0.96 ± 0.02	0.96 ± 0.02
TA	0.92 ± 0.09	0.96 ± 0.03	0.96 ± 0.02
GAS	0.93 ± 0.11	0.97 ± 0.02	0.98 ± 0.02
Lag time, %			
BB	1.3 ± 1.8	1.8 ± 2.9	0.4 ± 2.6
TB	0.4 ± 0.5	1.4 ± 2.3*	0.5 ± 1.5
DA	2.4 ± 1.3*	2.3 ± 1.5*	−0.1 ± 0.4
DP	1.6 ± 2.6	1.1 ± 2.5	−0.4 ± 1.5
PM	0.9 ± 4.1	1.9 ± 3.1	0.8 ± 1.5
LD	2.8 ± 3.5*	2.5 ± 3.7*	−0.3 ± 0.7
RA	−3.0 ± 10.1	−2.9 ± 10.3	0.5 ± 1.7
ES	−1.1 ± 7.4	−1.4 ± 7.3	−2.1 ± 5.4
RF	5.0 ± 7.8*	5.1 ± 7.9	0.3 ± 0.7
BF	2.6 ± 2.7*	3.1 ± 3.1*	0.6 ± 1.2
TA	4.5 ± 10.1	4.9 ± 10.0	0.4 ± 0.7
GAS	6.4 ± 8.7*	7.4 ± 8.5*	0.8 ± 1.6

All data presented as Mean ± SD. A negative value for lag indicates a backward shift compared to BE cycle or BABH cycle. An asterisk indicates a significant difference from zero, *p* < 0.05.

**Figure 3:**
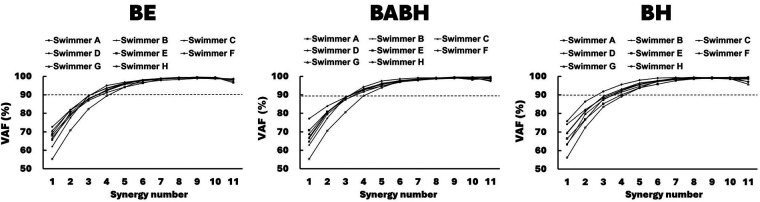
Results of VAF for each number of muscle synergies for each participant.

**Figure 4:**
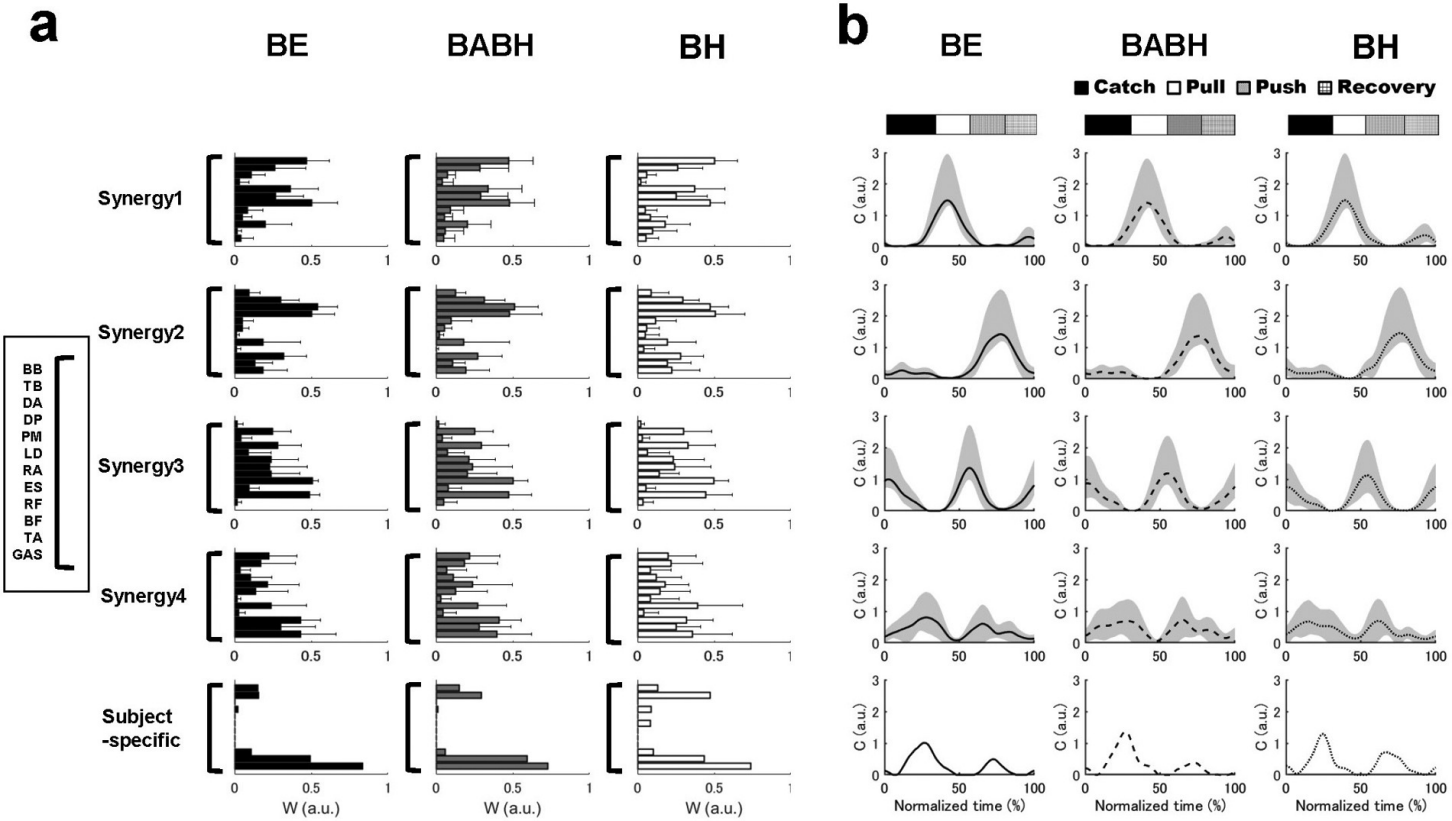
Results of the W in the extracted synergies **(a)** and result of C in the extracted synergies **(b)**. In **(a)**, the black bars show the results in the BE cycle, the gray bars show the results in the BABH cycle, and the white bars show the results in the BH cycle. In **(b)**, the solid lines show the results in the BE cycle, the dash lines show the results in the BABH cycle, and the dotted lines show the results in the BH cycle. The horizontal bar graphs show the timing of the average arm stroke phases in each cycle.

Figure 3 shows the VAF results of the muscle synergy analysis. Based on this result, we determined that the number of synergies in the BE cycle was four for the 7 swimmers and five for the 1 swimmer, that the number in the BABH cycle was four for the 7 swimmers and five for the 1 swimmer and that the number in the BH cycle was three for the 1 swimmer and four for the 6 swimmers and five for the 1 swimmer. Figures 4a,b show the results of W and C for the BE, BABH and BH cycles. The sorted four synergies extracted from all participants were defined as Synergy 1 (*n* = 8), 2 (*n* = 8), 3 (*n* = 7), and 4 (*n* = 8), and the fifth synergy observed from one swimmer was categorized as a subject-specific synergy. In Synergy 1, the main contributors were BB, PM, and RA in the BE cycle; BB, PM, and RA in the BABH cycle; and BB, PM, and RA in the BH cycle. In Synergy 2, the main contributors were TB, DA, DP, and BF in the BE cycle, TB, DA, and DP in the BABH cycle, and DA and DP in the BH cycle. For Synergy 3, the main contributors were RF, and TA in the BE cycle; RF and TA in the BABH cycle; and RF and TA in the BH cycle. In Synergy 4, the main contributors were BF and GAS in the BE cycle, BF, and GAS in the BABH cycle, and ES, BF, and GAS in the BH cycle. Table 4 lists the SP results of W for the five synergies. Table 5 lists the results of *r*_max_ and lag time for C in the five synergies. The results of the one-sample test for the lag time showed that the drive timing of Synergy 1 and 4 in the BH cycle became earlier than those in the BE cycle (both *p* = 0.03, both *r* = 0.78).

**Table 4:** Results of the SP for the W of the extracted muscle synergies.

Synergy name	SP (*r*)		
	BE vs. BABH	BE vs. BH	BABH vs. BH
Synergy1	0.97 ± 0.03	0.94 ± 0.04	0.93 ± 0.04
Synergy2	0.97 ± 0.04	0.90 ± 0.09	0.92 ± 0.13
Synergy3	0.97 ± 0.02	0.94 ± 0.06	0.95 ± 0.07
Synergy4	0.96 ± 0.05	0.86 ± 0.18	0.79 ± 0.29
Subject-specific	0.98	0.94	0.96

All data presented as Mean ± SD.

**Table 5:** Results of the waveform similarity (r_max_) and lag time for the C of the muscle synergies.

Variable, unit	BE vs. BABH	BE vs. BH	BABH vs. BH
*r*_max_			
Synergy 1	0.98 ± 0.02	0.97 ± 0.02	0.98 ± 0.02
Synergy 2	0.98 ± 0.03	0.97 ± 0.01	0.98 ± 0.01
Synergy 3	0.98 ± 0.01	0.95 ± 0.02	0.95 ± 0.04
Synergy 4	0.94 ± 0.11	0.92 ± 0.09	0.95 ± 0.04
Subject-specific	0.98	0.95	0.94
Lag time, %			
Synergy 1	1.0 ± 2.1	2.5 ± 2.4*	1.6 ± 2.7
Synergy 2	0.8 ± 0.9	0.8 ± 1.5	−0.1 ± 2.2
Synergy 3	0.3 ± 1.0	0.7 ± 1.0	0.4 ± 1.3
Synergy 4	0.3 ± 2.3	6.1 ± 8.5*	3.1 ± 7.1
Subject-specific	0.0	2.0	2.0

All data presented as Mean ± SD. A negative value for lag indicates a backward shift compared to the slower trial. An asterisk indicates a significant difference from zero, *p* < 0.05.

## 4 Discussion

This study aimed to clarify the effect of different breathing frequencies with breath-holding on muscle activity and coordination during butterfly swimming in competitive swimmers. The main findings revealed that the activity timing for some muscles became earlier in the BABH and BH cycles than in the BE cycle and that the drive timing for two muscle synergies became earlier in the BH cycle than in the BE cycle. These results suggest that muscle activity and synergies during butterfly swimming are affected by breathing action and different breathing frequencies.

The kinematic results showed that V_mean_, V_max_, V_min_, SR, SL and dV did not change between the three different cycles (Table 2). Therefore, it was suggested that our participants maintained their performance while performing breathing actions and different breathing frequencies. However, there was a large effect size in the result in V_max_ and the average V_max_ in the BH cycle was higher than that in the BE and BABH cycles (Table 2). Some studies have indicated that butterfly swimming without breathing may have a higher performance than swimming with breathing action (1, 3–5). Hence, it was considered that the butterfly stroke movement with breath-holding improved the maximum swimming velocity for some swimmers. Furthermore, the push and recovery phases became longer in the BH cycle (Table 2). Overall, the changes in the phase structure of arm stroke related to the BH cycle seemed to reduce the time gap between the start of the arm stroke and the start of the first upward kick and to increase the time gap between the start of the arm recovery and the start of the second upward kick. These changes between the breathing and breath-holding cycles were consistent with the results of a previous study (4). In addition, it was observed that the DK-1st phase exhibited a tendency to decrease in the BABH cycle compared to other cycles (Table 2). From this result, it was suggested that different breathing frequencies cause slight changes in the phase structure of leg movement.

Some studies on butterfly swimming have reported the activity patterns of individual muscles of the upper limb, trunk, and lower limb during a cycle (9, 21–23). Our results for the activity patterns of individual muscles are similar to those of previous studies. The timing of activity for TB, DA, LD, BF, and GAS became earlier in the BH cycle than in the BE cycle (Table 3). These changes in activity timing correspond well with the changes in the arm phase structure between the BE and BH cycles. Interestingly, the activity timing for DA, LD, RF, BF and GAS also became earlier in the BABH cycle than in the BE cycle (Table 3) while the activity timing for all the muscle did not change between the BABH and BH cycles. Therefore, it was suggested that different breathing frequencies may change the activity timing for some muscles. The aEMG for all muscles did not change between the three different cycles (Figure 2b). From this result, it was suggested that the intensity of activity for all the muscles analyzed in this study remained across the different breathing frequencies or with and without frontal breathing action, and one of our hypotheses was not approved. Alves et al. (3) indicated that elite swimmers could reduce unnecessary movements of the trunk and lower limbs during frontal breathing action. Therefore, it was considered that the muscle activity did not increase by the frontal breathing action because our participants were national-level competitive swimmers.

The W values of all synergies had high similarity between the three different cycles (Table 4). From this result, it was suggested that our participants achieved the same synergy-based movement across the three different cycles, and our hypothesis was not supported. Yamakawa et al. (9) reported that less-skilled swimmers coordinated their arm-catch and arm-push movements with their downward kicks to prevent the lower limb from sinking in response to the arm-catching movement or the breathing action, whereas skilled swimmers did not. In addition, elite swimmers are able to perform breathing movements without compromising their swimming speed (3). Therefore, it has been speculated that our participants achieved both movements with and without the breathing action using the same muscle synergies to effectively perform propulsive action. In contrast, the results of the C showed that the drive timing of Synergies 1 and 4 in the BH cycle became earlier than in the BE cycle (Table 5), suggesting that our participants changed the temporal component of the synergies between the BH and BE cycles. Based on the values of W and C for Synergies 1 and 4, we judged that Synergy 1 is a synergy acting on the arm-pull movement and Synergy 4 is a synergy acting on the first and second upward kicks. The changes in the temporal component for Synergies 1 and 4 corresponded well with the changes in the activity timing of some muscles and the changes in the arm phase structure. Barbosa et al. (5) reported that during the out-sweep phase of the breath-holding cycle the horizontal hand velocity was higher and the vertical displacement of the hands was smaller than those in the cycles with frontal breathing action, and they concluded that propulsive force may be generated earlier in the stroke during non-breathing phases. Therefore, it was considered that the changes in the temporal components of these synergies caused the changes in the activity timing of some muscles, resulting in moving arm and leg movements earlier. Furthermore, the results of the C showed that the drive timing of all synergies was consistent between the BABH and BH cycles. From these results, it was speculated that changes in the temporal components of the muscle synergy observed in this study were more influenced by different breathing frequencies than by breathing action. Therefore, researchers should consider the effect of different breathing frequencies when analyzing muscle activity or muscle synergy during butterfly swimming.

In terms of practical implications, our results emphasize the importance of learning the timing of arm and leg movements to achieve better breathing action. The neural basis of muscle synergy related to W and C has been elucidated in recent neurophysiological studies. In a review of the neural basis of muscle synergies (24), it indicated that the organizing (i.e., W) and driving (i.e., C) of the muscle synergies may originate from neurons at different levels that may be anatomically localized to different regions of the central nervous system. Accordingly, the different temporal characteristics of muscle synergies may promote different motor learning in the central nervous system region associated with driving C. Therefore, we recommend that athletes should train with the same breathing pattern that they will use during the race. Furthermore, one swimmer, who reduced the number of muscle synergies in the BH cycle, had the largest difference in the V_mean_ between the BE and BH cycles, and it was observed that this swimmer increased the cooperation between the upper and lower limb muscles in the BH cycle from the results of the W in Synergy 1 and 2. As in this case, there is a possibility that motor control will change dramatically in some swimmers whose performance has changed significantly between the breathing and non-breathing cycles. Thus, muscle synergy analysis can be useful for assessing changes in motor control during different swimming movements.

This study has some limitations. As this study did not measure neck muscle activity because these muscles are not directly involved in generating propulsion, it was possible that there were changes in muscle activity and muscle synergy associated with changes in head movements. Future studies should examine the additional muscles involved in this composite technique. We attached a marker and wireless EMG devices to the swimmers’ bodies. As the attachment of this experimental equipment affects movement and water resistance (25), the swimming movement during the trials included its effects. All participants of this study were female national-level swimmers. A kinematic study of butterfly swimming (12) reported significant differences in the swimming velocity and arm-to-leg coordination based on sex and skill level. Although a previous study reported that there were no differences between elite-level male and female swimmers, differences in sex and achievement levels may have affected our results. In the experimental tasks of this study, participants were asked to perform butterfly swimming at different breathing frequencies. Hence, it is possible that they performed the tasks at unfamiliar frequencies. Of the participants, two preferred to breathe once per stroke and six preferred to breathe once every two strokes. Therefore, the familiarity of breathing frequency may have influenced our results. Moreover, participants were also asked to perform butterfly swimming with sprint pace using frontal breathing action. In butterfly swimming, lateral breathing style tends to produce a flatter swimming movement than frontal breathing style (26). At slower swimming speeds, duration of arm catch increases (14), emphasizing undulating movements and eliminating differences in the timing of arm and leg movements between breathing and breath-holding cycles (4). Therefore, the results of this study are only considered applicable to frontal breathing action and sprint pace. Finally, the limitations also included the small sample size, the participants’ different stroke specialties, and the different pool conditions compared to those in the competition.

## 5 Conclusion

In national-level female swimmers, the different breathing frequencies with breath-holding did not affect the butterfly swimming speed, whereas the arm and leg phase structure changed in the breath-holding cycle. The intensity of activity in all the muscles remained consistent throughout the three cycles. The spatial components of all the synergies were very similar across the three different cycles, suggesting that the swimmers achieved the same synergy-based movements in these cycles. In the breath-holding cycle, the activity timing of the five muscles and the drive timing of the two muscle synergies changed earlier compared to the cycle in which the swimmers breathe with each stroke. In contrast, the activity timing for all the muscles and the drive timing for all the muscle synergies did not change between the breathing and breath-holding cycles when swimmers performed cycles alternating between breathing and breath-holding. These results suggest that standardising breathing frequency is more important than considering with and without breathing actions when the researchers analyse the muscle activity during butterfly swimming.

## Data Availability

The original contributions presented in the study are included in the article/Supplementary Material, further inquiries can be directed to the corresponding author.
